# Intensified job demands, stress of conscience and nurses' experiences during organizational change

**DOI:** 10.1177/09697330211006831

**Published:** 2021-08-10

**Authors:** Mikko Heikkilä, Mari Huhtala, Saija Mauno, Taru Feldt

**Affiliations:** University of Jyväskylä, Finland; University of Jyväskylä, Finland; Tampere University, Finland; University of Jyväskylä, Finland

**Keywords:** Healthcare, intensified job demands, nurses, organizational change, stress of conscience, work intensification

## Abstract

**Background:**

Nurses frequently face ethically demanding situations in their work, and these may lead to stress of conscience. Working life is currently accelerating and job demands are intensifying. These intensified job demands include (1) work intensification, (2) intensified job-related planning demands, (3) intensified career-related planning demands, and (4) intensified learning demands. At the same time, many healthcare organizations are implementing major organizational changes that have an influence on personnel.

**Aim:**

The aim of the study was to investigate the association between intensified job demands and stress of conscience, and whether their association is moderated by organizational change experiences among nurses. Experiences of organizational change may expose employees to stress of conscience or serve as a buffer because employees appraise, involve, and cope with changes differently.

**Research design:**

Questionnaires measuring stress of conscience, intensified job demands, and organizational change experiences were completed by nurses (n = 511) in a healthcare district undergoing a major organizational change.

**Ethical considerations:**

Throughout, the study procedures were implemented according to the guidelines of the Finnish National Board on Research Integrity and the 1964 Helsinki Declaration and its later amendments. According to the Finnish regulations, because participation was voluntary, informed consent was requested, and participants were advised of their right to withdraw from the study at will. No permission from an ethics committee was necessary.

**Findings:**

Work intensification and personal worry considering organizational change were associated with more severe stress of conscience among nurses. Nurses’ experiences of managements’ competent handling of organizational change buffered the association between work intensification and stress of conscience.

**Conclusions:**

During organizational changes, management may alleviate nurses’ stress of conscience by proper communication and support procedures.

## Introduction

Healthcare is primarily a moral endeavor because its aim is to do good or the right thing for patients.^1^ However, healthcare personnel may not be able to constantly provide the good care they want to, and this causes ethically difficult situations, which, in turn, can result in stress caused by a troubled conscience, that is, *stress of conscience.*^1,2^ The concept of stress of conscience has been related to situations in which employees cannot follow their consciences at work and are not able to deal with or solve ethical problems, for example, when nurses have not enough time to provide the care the patient needs or when they must lower their aspirations to provide good care.^1^ Such situations may cause feelings that one has not done enough, which, in turn, may result in stress caused by a troubled conscience.^2^ Healthcare personnel encounter ethically difficult situations frequently, while according to many researchers contemporary working life is under acceleration and intensification, creating *intensified job demands* (IJDs).^3–5^ This development includes demands for a more rapid work pace, self-directed planning and decision-making at work, keeping oneself attractive to the labor market, and a constant need to learn new skills and acquire new knowledge at work.^4,5^ IJDs may challenge nurses’ opportunities to maintain basic nursing values^6^ and, thus, lead to stress of conscience. At the same time, many organizations—including healthcare organizations—encounter major organizational changes.^7^ Employees’ experiences of organizational change have multiple facets. Organizational change can lead to higher levels of job stressors and demands,^8^ increased workload,^9^ and cause uncertainty and worry.^10^ Organizational structures may also contribute to work overload that forces nurses to compromise on basic nursing values, leading to ethical dilemmas.^6^ Thus, experiences of organizational change come at a price and can be seen as job stressors requiring extra effort over and above the other job stressors inherent in healthcare^8,11^ and may moderate the association between IJDs and stress of conscience. Despite constantly intensifying working life and organizational changes in healthcare organizations, we lack research evidence on how these phenomena are experienced from an ethical perspective, here, regarding stress of conscience among healthcare personnel.

The target group of this study were nurses doing care work. Nurses are the front liners and possibly the most vulnerable employee group in healthcare to be affected by stress of conscience because they have to deal daily with challenges in the attempt to provide good care, though at times setbacks are inevitable.^1,2^ IJDs may generate risks to nurses’ health and well-being, for example, by increasing the stress of conscience and burnout scores.^12,13^ Furthermore, organizational changes may cause nurses extra demands and stress.^8,14^

Our first aim was to investigate whether IJDs are associated with stress of conscience among nurses. The second aim was to investigate whether experiences of organizational change are associated with stress of conscience. The third aim was to investigate the potentially moderating effect of experiences of organizational change in the association of IJDs with stress of conscience

## Definitions of the concepts

*Stress of conscience* is defined as “a product of the frequency of the stressful situation and of the perceived degree of troubled conscience as rated by healthcare personnel themselves” (p. 636).^1^ Glasberg^1^ defines conscience as representing core values on how we have to be and act to retain our moral integrity and peace of mind. Stress of conscience has been related to situations in which employees cannot follow their conscience at work and are not able to deal with or solve ethical problems, for example, when they have not enough time to provide the care the patient needs or when they must lower their aspirations to provide good care.^1^ Such situations may cause feelings that one has not done enough, which, in turn, may result in stress caused by a troubled conscience.^2^

When examining factors characterizing and constructing stress of conscience, one Finnish study showed that the most severe stress of conscience was reported by healthcare personnel because of not having enough time to provide good care.^15^ The results were similar to those of the original Swedish stress of consciences study.^2^ There is evidence of an association of healthcare professionals’ stress of conscience level with their colleagues’ stress of conscience level.^16^ Experience of control over situations at work may protect nursing staff in psychiatric care against stress of conscience, while a high level of sense of moral burden and perceptions of the angry and aggressive behavior of patients may increase stress of conscience.^17^ However, earlier research has not taken into consideration the effect of the acceleration of society on stress of conscience. IJDs in health care may lead to situations where nurses cannot achieve their ethical standards, which may in turn increase stress of conscience. Our study fills this gap in the research.

*IJDs* include demands for a more rapid working pace, increased self-directed planning and decision-making at work, keeping oneself attractive to the labor market, and a constant need to acquire new skills and new knowledge at work.^4^ IJDs are rooted in the acceleration of modern societies manifesting in the interconnected acceleration of technology, social change, and pace of life (see more^5^). The acceleration of technology, social change, and pace of life fuels each other in an interconnected cycle, which is expected to accelerate working life.^5^ Here, we adopt the model proposed by Kubicek et al.,^4^ where five different aspects of IJDs are defined: (1) work intensification, (2) intensified job-related planning and decision-making demands, (3) intensified career-related planning and decision-making demands, (4) intensified knowledge-related learning demands, and (5) intensified skill-related learning demands.

All five dimensions of IJDs have been found to be associated with burnout, of which work intensification has explained the largest amount of variance among white-collar employees.^18^ The amount of research on IJDs is increasing.^4,18–21^ Findings from these studies show that work intensification has generally been related to increasing work strain. In healthcare, work intensification has been associated with disengagement and emotional exhaustion.^12^ Among nurses, work intensification has been associated with increased stress and decrease in job satisfaction.^22^ We contribute to the research on IJDs by investigating how IJDs are associated with nurses’ well-being in a specific context: a healthcare organization undergoing a major organizational change.

Organizational change means a major change in an organization’s structure, which affects each employee more or less.^23^ We focus on *experiences of organizational change* because employees appraise, involve, react, adopt attitudes, and cope with changes differently.^24–27^ In successful organizational change management, management’s change communication and adequate information for personnel determine much of the outcome of the change, and when rightly done, successful change management can enhance the work community and trust in management.^27,28^ Mutual trust and consultation between employees and higher management are crucial for a major change.^26,29^ If management merely announces change information and instructions, personnel may perceive their opportunities to influence changes to be low.^7^ Management’s support for employees also enhances well-being and the likelihood of a successful change.^30^

Another aspect of organizational change for the employees is their own involvement and actions taken during the change process. Hospital employees with autonomy in their work have been found to be more proactive in an organizational change.^31^ Employees’ willingness to get involved and take action has been shown to reduce cynicism toward organizational change.^32^ Additional aspects of organizational change are increased uncertainty and worry among employees because organizational change leads to novel experiences^8^ and has an association with job insecurity.^10,33^ Organizational change experiences and IJDs may also co-occur and reinforce each other. For example, organizational change led to work intensification in Canadian teaching hospitals.^22^ Therefore, it is important to study the combined effects of IJDs and organizational change experiences.

In our discussion, we adopt Kanter’s structural empowerment theory as a point of view to explain how some of the nurses’ management of organizational change experiences affects their stress of conscience. Structural empowerment is enhanced by giving employees access to information, resources, and support.^34,35^ Empowered employees are more committed to the organization and cope better with job demands.^34,35^ Management’s organizational change actions may have qualities comparable to those of structural empowerment. Therefore, we suspect that empowerment of nurses may have an effect on their experience of stress of conscience.

## Aims and assumptions

We had three major aims in this study. *The first aim* was to investigate whether IJDs are associated with stress of conscience among nurses in a healthcare district in Finland. Stress of conscience in healthcare has to do with employees’ perceptions that they are unable to achieve the ethical goals of working with patients—specifically to provide good care and protect patients from harm. This may be impaired due to numerous reasons, for example, lack of resources and time, or when contravening regulations while acting according to their moral beliefs.^2^ Stress of conscience has its roots in Lazarus and Folkman’s transactional theory of psychological stress,^2^ which defines stress as a result of interaction between the individual and the environment.^36^ Stress appears when an individual encounters a situation where the demands or expectations of the environment exceed an individual’s personal resources. Thus, following the idea of this theoretical framework, we assumed that IJDs are associated with more severe stress of conscience.

*The second aim* was to investigate whether experiences of organizational change are associated with stress of conscience among nurses. For employees, such experiences may be stressors or protective elements and are colored by their appraisal of the change.^25,36^ Rafferty and Griffin^8^ suggest that uncertainty is a critical cognitive appraisal resulting from change. However, this perception can be altered by proper communication and information shared by management.^8,37^ Because of these elements, we assumed that experiences of organizational change are associated with stress of conscience, so positive change experiences are associated with less severe stress of conscience and negative change experiences with more severe stress of conscience.

*The third aim* was to investigate the potentially moderating effect of experiences of organizational change in the association of IJDs with stress of conscience. Experiences of organizational change may be stressors or buffers. For example, organizational change may, because of insufficient information, consultation, and support,^10^ increase psychological uncertainty,^8^ which may increase employees’ fatigue and exhaustion.^38^ This may impair the quality of care,^39^ which may increase stress of conscience among nurses as they perceive, for example, that they lower their aspiration to provide proper care. However, supervisor’s support has been shown to buffer the association between change stressors and exhaustion.^11^ Hence, we assumed that management’s support may reduce stress of conscience among nurses because if management has taken the nurses’ views into consideration, it may, for example, alleviate their psychological uncertainty and fatigue and, thus, help to maintain adequate care quality and therefore not increase stress of conscience. Consequently, we assumed that experiences of organizational change may have a moderating effect on the association between IJDs and stress of conscience. By examining these moderating effects, we hope to find helpful implications for healthcare organizations during organizational change to prevent stress of conscience among personnel.

## Participants and procedure

Participants were nurses doing care work from a large municipal healthcare organization in Finland, which consisted of a central hospital and several health centers. The organization was undergoing a major organizational change, which included moving from a traditional organizational model to a patient-centered hospital model and relocating the hospital services to a new unit. The researchers’ electronic questionnaire was sent via the organization’s representative as an invitation email to all employees in September 2019, before COVID. Two reminders were sent by the organization’s representative in October 2019. The invitation and reminders included a description of the project, information about voluntary participation, confidentiality, collection and use of personal data, and the link to the electronic survey. The respondents gave their informed consent to participate by responding to an accompanying item before being able to continue further in the survey. The participants’ responses came back to researchers anonymously. Participants numbered 1024 (response rate, 25%). This study focused on nurses (n = 511), who represented 73% of the whole patient care personnel in the sample. The majority of the nurses were women (91%), and the largest age group was 41–45 years old. The most typical nurse occupations were registered nurse, midwife, and X-ray nurse. More than two-thirds (70%) of the nurses worked in shifts.

## Ethical considerations

Throughout, the study procedures were implemented according to the guidelines of the Finnish National Board on Research Integrity and the 1964 Helsinki Declaration and its later amendments or comparable ethical standards. According to the Finnish regulations, no review from an ethics committee was necessary, and because participation was voluntary, informed consent was requested and participants were assured that they were free to withdraw from the study at any time. Furthermore, no personal health data were collected, nor was precise age elicited; only a structured age distribution was used. Participants were assured of confidentiality that the data would be used in congruence with European Union General Data Protection Regulation.

## Measurers

*Stress of conscience* was measured with the Stress of Conscience Questionnaire (SCQ).^2^ In the SCQ, frequency of situations causing stress of conscience was elicited first and then the magnitude of stress (later stress level) caused by these situations. Usually, stress of conscience is measured by multiplying its frequency by the stress magnitude;^2,13^ however, we wanted to separate the dilemma frequency from the stress magnitude to find out which one, frequency or magnitude, affects stress of conscience more among nurses.

*IJDs* were measured with the Intensification of Job Demands Scale (IDS).^4^ The scale evaluates intensification of job demands in the last 5 years with five sub-scales: intensified work demands, intensified job-related planning demands, intensified career planning demands, intensified knowledge-related learning demands, intensified skill-related learning demands. Intensified knowledge-related learning demands and intensified skill-related learning demands scales were combined into intensified learning demands because intensified knowledge-related learning demands and intensified skill-related learning demands correlated highly with each other (.81, p < .001). The combined intensified learning demands scale has been used in previous research for the same reason.^18^

*Experiences of organizational change* were measured with the Meaningful Organizational Change Scale.^23^ The scale measured three dimensions: (1) how employees experienced management action (informing, considering, and giving support to personnel) in the change (organizational change management), (2) employees’ own actions (changed their work to be more meaningful, used available support, and developed know-how) considering the change (organizational change personal action), and (3) how worried the employees were about their ability to function in the altered situation (organizational change personal worry). In the original scale, the organizational change personal worry question is included among the organizational change personal action questions, but then the reliability coefficient of the dimension was low (Cronbach’s alpha = .34). Without the personal worry item, the organizational change personal action dimension had good reliability (see Table 1). We decided to take organizational change personal worry as a separate item because it reveals employees’ point of view from a different and meaningful perspective on their experiences of organizational change.

**Table 1.** Summary table of measures and descriptive figures (M, SD) among nurses (n = 511) concerning nine sub-scales.

Scale	Alpha coefficients	M (SD)	No. of items	Scale rangea	Example items
SC frequency	.81	2.91 (0.94)	7	1–6/F	“How often do you lack the time to provide the care the patient needs?”
SC stress level	.86	3.65 (1.14)	7	1–6/L	“Does this give you a troubled conscience?”
WI	.91	3.86 (1.00)	5	1–5/L	“In the last five years even more work has to be accomplished by fewer and fewer employees”
JP	.83	3.52 (0.86)	5	1–5/L	“In the last five years decisions about the work had to be made more and more often without being able to discuss with a superior.”
CP	.78	3.59 (0.92)	3	1–5/L	“In the last five years one has been increasingly required to maintain one’s attractiveness for the job market, e.g., through further education, networking.”
LD	.90	4.13 (0.70)	6	1–5/L	“In the last five years one has to update one’s knowledge level more frequently.”
OC management	.84	2.44 (0.84)	4	1–5/L	“The management of the organization has informed clearly about the aim of the change .”
OC personal action	.66	3.43 (0.72)	3	1–5/L	“I have actively utilized the benefits that the change offers to make my work more meaningful.”
OC personal worry	–	3.11 (1.28)	1	1–5/L	“I have been worrying, how I can act in the changed situation.”

SC: stress of conscience; WI: work intensification; JP: intensified job planning demands; CP: intensified career planning demands; LD: intensified learning demands; OC: organizational change experiences; F: frequency-based scale construct; L: Likert-type scale.

Scale range: Higher score indicates more of the given construct.

*Control variables* in the regression analysis were gender (female/male), age (nine categories), and work shift (daytime work/shift work). Gender and work shift have been found to have an association with stress of conscience; thus, they were chosen as control variables.^1^ Age has not been found to be associated with stress of conscience, but it has been shown that older employees experience more IJDs.^19^ Summary of measures, means, and standard deviations of variables among nurses are described in Table 1.

## Statistical analysis

Statistical analyses were performed with Statistical Package for Social Sciences (SPSS) (version 25). First, we conducted a descriptive analysis in relation to nurses’ characteristics. Pearson’s correlations were used to ascertain the relations between variables. Then, hierarchical regression analysis was used to determine the main effects of IJDs and experiences of organizational change on the outcomes of stress of conscience. We then conducted hierarchical moderated regression analyses with interaction terms to examine the moderating effects of the aspects of experiences of organizational change (organizational change management, organizational change personal action, and organizational change personal worry) in the relationship between IJDs (work intensification, intensified job planning demands, intensified career planning demands, and intensified learning demands) and the outcomes of stress of conscience (frequency and stress level). Finally, we calculated simple slope tests for the significant interaction effect.

## Results

Correlations between predicting variables and outcomes among nurses are described in Table 2.

**Table 2.** Intercorrelations of the study variables for nurses (n = 511).

Variables	1	2	3	4	5	6	7	8	9	10	11	12	13
1. SC frequency	–												
2. SC stress level	.43***	–											
3. Gender (f/m)	–.05	–.24***	-										
4. Age	–.11*	–.02	–.04	-									
5. Work shift (d/s)	.21***	.12**	–.01	–.21***	–								
6. WI	.40***	.33***	–.03	–.01	.13**	–							
7. JP	.24***	.21***	–.09*	.10*	–.16***	.40***	–						
8. CP	.18***	.15***	–.13**	–.05	–.13**	.31***	.67***	–					
9. KL	.20***	.21***	–.13**	.09*	–.02	.27***	.47***	.41***	–				
10. SL	.25***	.20***	–.05	.13	.07	34.***	.38***	.31***	.81***	–			
11. OC management	–.26***	–.15***	–.05	.01	–.04	–.31***	–.23***	–.19***	–.11**	–.13**	–		
12. OC personal action	–.07	–.01	.01	–.03	–.13**	–.04	.11*	.25***	.15**	.06	.21***	–	
13. OC personal worry	.32***	.26***	–.09	.00	.15**	.16***	.09*	.01	.12**	.15***	–.18***	–.13***	–

Gender (f/m): female/male. Age was measured with nine age groups: 1 = 25 years or under, 2 = 26–30 years, 3 = 31–35 years, 4 = 36–40 years, 5 = 41–45 years, 6 = 46–50 years, 7 = 51–55 years, 8 = 56–60 years, and 9 = >60 years. Work shift (d/s): day/shift work; SC: stress of conscience; WI: work intensification; JP: intensified job-related planning; CP: intensified career planning; KL: intensified knowledge demands; SL: intensified skill demands; OC: organizational change.

* p < .05; **p < .01; ***p < .001.

Considering stress of conscience frequency, we report here exclusively the findings from the third step of the regression analysis as these take into account the power of IJDs and experiences of organizational change when all variables are included in the model. Among nurses (Table 3), 28% variance in stress of conscience frequency was explained by shift work, work intensification, and worry about organizational change, whereas older age and positive experiences of organizational change management contributed negatively to frequency of stress of conscience. These findings mean that older age and good experiences of change management related to less severe stress of conscience among nurses.

**Table 3.** Results of hierarchical regression analyses of IJDs and organizational change experience and significant interaction terms on stress of conscience frequency and stress of conscience stress level among nurses (n = 511) in a large health care organization.

Model	Step 1.		Step 2.		Step 3.		Step 4.	
Independent variables	SC frequency	SC stress level	SC frequency	SC stress level	SC frequency	SC stress level	SC frequency	SC stress level
Variables	β	β	β	β	β	β	β	β
Gender (f/m)	–.18	–.82***	–.09	–.75***	–.05	–.72***	–.05	–.72***
Age	–.07	.00	–.09*	–.02	–.10*	–.02	–.10*	–.02
Work shift (d/s)	.42***	.25*	.35***	.18	.28**	.14	.28**	.18
WI			.31***	.25***	.26***	.23***	.26***	.24***
JP			.13*	.09	.09	.07	.09	.07
CP			–.02	–.04	.00	–.03	.00	–.04
LD			.09	.09	.08	.07	.08	.08
OC management					–.10*	–.04	–.10*	–.04
OC personal action					–.02	.04	–.02	.03
OC personal worry					.22***	.18***	.22***	.17***
WI × OC management							–	–.09*
Overall *R*^2^	.05***	.07***	.22***	.18***	.28***	.21***	.28*	.22*
Δ*R*^2^	–	–	.17***	.11***	.06***	.03***	.00*	.01*

β: standardized beta-coefficient. Gender (f/m) = female/male. Age was measured with nine age groups 1 = 25 years or under, 2 = 26–30 years, 3 = 31–35 years, 4 = 36–40 years, 5 = 41–45 years, 6 = 46–50 years, 7 = 51–55 years, 8 = 56–60 years and 9 = >60 years. Work shift (d/s): day/shift work, SC: stress of conscience; WI: work intensification; JP: intensified job-related planning; CP: intensified career planning; KL: intensified knowledge demands; SL: Intensified skill demands; OC: organizational change.

* p < .05; **p < .01; ***p < .001.

**Figure 1.**
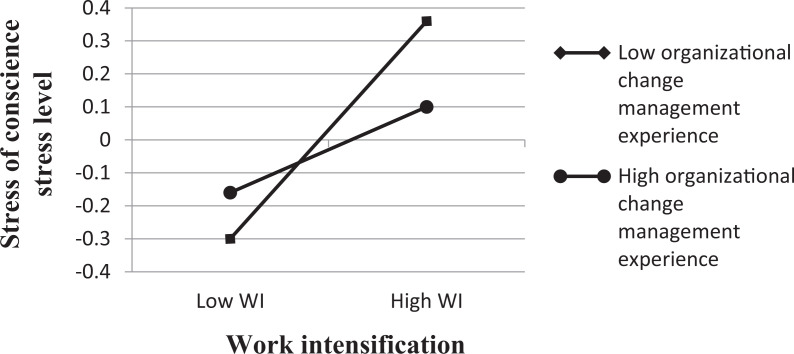
Interaction effect of work intensification × organizational change management on stress of conscience stress level among nurses.

Of the stress of conscience level, the “work intensification × experience of managements actions in organizational change” interaction term explained a significant amount of variance (1%) (see Table 3). More precisely, nurses had the lowest stress of conscience frequency when they experienced low work intensification (−1 SD below mean) regardless of their experience of management’s actions in organizational change (see Figure 1). However, nurses reporting high levels of work intensification (+1 SD above mean) and low scores on experience of management’s actions in organizational change reported significantly higher levels of stress of conscience level than did nurses with positive experience of management’s actions in organizational change. According to simple slope analysis, among nurses with poor experience of management’s actions in organizational change (−1 SD), the association between stress of conscience level was stronger (β = .33, p < .001) than among those with good experience of management’s actions in organizational change (β = .16, p < .05). The main effect of work intensification is also evident in Figure 1: the higher the work intensification, the higher the level of stress of conscience.

## Discussion

The main aim of this study was to explore among nurses, for the first time, the association between experiences of IJDs and stress of conscience. The second aim was to ascertain whether this association was moderated by organizational change experiences. The study was conducted in a healthcare district undergoing a major organizational change. Of the IJDs investigated, work intensification was found to be a risk factor for nurses’ stress of conscience in our quantitative study. Similar results have been found in the qualitative studies by Pomare et al.^27^ and Smollan.^10^

The results showed that work intensification was associated with higher frequency and more severe level of stress of conscience. This may be because work intensification in healthcare is connected to compromised patient safety and missed care, for example, nurses need to rationalize care and hence not provide all care they should.^40^ Work intensification is also associated with adverse events happening to patients, for example, because of communication gaps among the personnel.^41^ Missed care and adverse events challenge nurses with ethical dilemmas.^42^ Meanwhile, nurses face increasing patient acuity, changing patient populations, and increasingly complex demands.^43^ Moreover, work intensification removed periods at work that facilitate recovery.^43^ Thus, nurses’ workload may exceed their personal resources and lead to stress^36^ and fatigue, which may further impair quality of care.^39^ Work intensification therefore may increase nurses’ stress of conscience as they perceive the consequences of the phenomenon in their daily work.

The results indicated that nurses’ worry about organizational change—meaning that nurses were worried about how they can act in the forthcoming changed situation—was associated with higher frequency of more severe stress of conscience and higher stress levels. Concern about organizational change may include anticipation of inadequate staffing and possible breakdown of collaboration because of a new workspace layout.^27^ These concerns may be combined with feelings of being uninformed about the change, being fatigued, and being under-staffed in the continuously changing environment.^27^ These worries may be interrelated and complex, for example, concern about inadequate staffing may be related to concerns about the new operating environment and patient care, which could impair teamwork and lead to work overload.^27^ Altogether, concern about organizational change and its complexity may be an exhausting additional stressor and may lead to stress and fatigue^27,39^ and, thus, may result, for instance, in situations where nurses cannot fulfill other’s expectations of their work and therefore feel increases in stress of conscience.

A further finding was that nurses’ experiences of management’s actions in organizational change were associated with lower frequency of stress of conscience. Nurses have few opportunities to influence organizational change and are therefore dependent on management’s actions in organizational change.^44^ When nurses feel that, during the change, management has informed them clearly about the goals of the change, taken the personnel’s viewpoints into consideration, ensured enough change support, and an immediate manager has discussed the change with their staff, it may be that nurses feel secure and less distressed about the organizational change.^11^ Security and less distress may help to maintain nurses’ well-being,^27^ and this may ensure proper care for patients.^39^ Proper care may lessen missed care and adverse events to patients,^40,41^ which may lower the nurses’ frequency of stress of conscience.

Nurses’ experiences of management’s competent information dissemination, consideration, and supporting personnel in organizational change buffered the association between work intensification and level of stress of conscience. Managements’ competent information dissemination, consideration, and support for personnel may be described as Kanter’s structural empowering working conditions^34^ Nurses’ empowerment may lead to better quality of care, effectiveness, and patient safety,^34,35^ which may counteract the challenges of work intensification, thereby reducing nurses’ stress of conscience level. In other words, during organizational change, management’s actions as structural empowerment counterbalance the negative effects of work intensification on nurses.

## Limitations

The study was based on self-report cross-sectional data, which does not allow interpretation of causality. Furthermore, the response rate of the survey was relatively low (25%), which may deduct the generalization of the results. Finally, it should be noted that the data were collected at the early preparatory stage of the organizational change. Therefore, the results do not indicate how the change process was implemented and how it succeeded from the viewpoint of the nurses.

## Recommendations for future research

Stress of conscience and ethical dilemmas are a topical issue in healthcare^6,45–47^ and need to be examined from different angles, for example, the exact mechanisms and process involved to work intensification that may increase stress of conscience. Follow-up research should therefore utilize a longitudinal mediator setting to establish whether the reasons leading to increasing stress of conscience, for example, are increasing pace of work and workload that impair adequate care or something else. Another aspect is that the organizational change was in its early stages, and we should examine afterward how the organizational change was experienced to ascertain what the associations between IJDs and stress of conscience will be when the situation has normalized.

IJDs and organizational changes and their associations with stress of conscience in nursing might be considered a worldwide phenomenon. Our study was conducted in Finland, therefore possibly limiting the depth of ethical issues that nurses may encounter elsewhere. For example, ethically challenging situations may arise from inadequate resource allocation, poor ethical training, and religious beliefs.^48,49^ Hence, we encourage further cross-cultural research to explore how IJDs and organizational change experiences explain stress of conscience. As ethical issues related to stress of conscience are a fluid cultural construct, it is questionable whether a similar stress of conscience questionnaire would be universally applicable. Furthermore, the questionnaire may impose a certain ethical viewpoint upon different cultures, which itself may be an ethical issue. However, cross-cultural research on the issue would bring more ethical implications to the field of nursing ethics and nurses working in different cultural contexts.

## Conclusion

The implications are that as management’s action in organizational change is crucial to nurses’ stress of conscience, attention needs to be paid to managers’ education, know-how, and communicative competence so that they can implement the change process openly, in a dialogical and supportive climate.^28^ In tackling stress of conscience directly, management can create opportunities for discussion with nurses, where they could share their experiences of situations which trouble their consciences in order to find ways to reconsider them and to deal with them.^50^ Discussing and being heard in issues to do with stress of conscience would help to modify one’s conscience to a kind of optimal middle way so that sensitivity to ethical issues is neither “too little or too much,” which again may help to cope when it is not possible to heed one’s conscience.^1^

Another possibility would be, for example, to organize mindfulness workshops which may help nurses to reduce their stress of conscience.^46^ An encompassing way for management to deal with stress of conscience in the midst of work intensification and organizational change would be to use structural empowerment of nurses.^35^ To reduce work intensification, and thus stress of conscience, the most effective way would possibly be to have sufficient nurses and assistant personnel to enable adequate care.^40^ This has to do with the most important implication that nurses should be given enough time to provide good care. Sufficient staffing reduces haste related to missed care, which has been reported as the most troubling situation causing stress of conscience to the nurses.^1,2,15^ It is an ethical choice to assign enough nurses to care for patients. We encourage ethical decision-making policy to balance expenditure cutting policies in nursing. The ethical decision-making should also consider nurses’ well-being in providing care.
